# Differences in metacognitive functioning between obsessive–compulsive disorder patients and highly compulsive individuals from the general population

**DOI:** 10.1017/S003329172300209X

**Published:** 2023-12

**Authors:** Monja Hoven, Marion Rouault, Ruth van Holst, Judy Luigjes

**Affiliations:** 1 Department of Psychiatry, Amsterdam UMC, University of Amsterdam, Amsterdam, the Netherlands; 2 Motivation, Brain & Behavior (MBB) Lab, Paris Brain Institute (ICM), Hôpital de la Pitié-Salpêtrière, Paris, France; 3 Département d’Études Cognitives, École Normale Supérieure, Université Paris Sciences & Lettres (PSL University), Paris, France

**Keywords:** confidence, obsessive-compulsive disorder, compulsivity, global confidence, analogue

## Abstract

**Background:**

Our confidence, a form of metacognition, guides our behavior. Confidence abnormalities have been found in obsessive–compulsive disorder (OCD). A first notion based on clinical case–control studies suggests lower confidence in OCD patients compared to healthy controls. Contrarily, studies in highly compulsive individuals from general population samples showed that obsessive–compulsive symptoms related positively or not at all to confidence. A second notion suggests that an impairment in confidence estimation and usage is related to compulsive behavior, which is more often supported by studies in general population samples. These opposite findings call into question whether findings from highly compulsive individuals from the general population are generalizable to OCD patient populations.

**Methods:**

To test this, we investigated confidence at three hierarchical levels: local confidence in single decisions, global confidence in task performance and higher-order self-beliefs in 40 OCD patients (medication-free, no comorbid diagnoses), 40 controls, and 40 matched highly compulsive individuals from the general population (HComp).

**Results:**

In line with the first notion we found that OCD patients exhibited relative underconfidence at all three hierarchical levels. In contrast, HComp individuals showed local and global *over*confidence and worsened metacognitive sensitivity compared with OCD patients, in line with the second notion.

**Conclusions:**

Metacognitive functioning observed in a general highly compulsive population, often used as an analog for OCD, is distinct from that in a clinical OCD population, suggesting that OC symptoms in these two groups relate differently to (meta)cognitive processes. These findings call for caution in generalizing (meta)cognitive findings from general population to clinical samples.

## Introduction

Humans have the ability to monitor and introspect on their own thoughts and cognitive processes, a process referred to as metacognition (Fleming, Dolan, & Frith, 2012). In our uncertain world, our metacognition, and in particular our sense of confidence, guides our behavior. The feeling of confidence helps us seek information (Balsdon, Wyart, & Mamassian, 2020; Desender, Murphy, Boldt, Verguts, & Yeung, 2019; Pescetelli, Hauperich, & Yeung, 2021; Rollwage et al., 2020), guides our learning (Cortese, 2022; Guggenmos, Wilbertz, Hebart, & Sterzer, 2016) and changes our mind (Stone, Mattingley, & Rangelov, 2022), especially when external feedback is lacking (Rouault, Dayan, & Fleming, 2019). There is great variability in how well humans are able to judge their own performance. Given the fundamental function of metacognition in guiding behavior, distortions in metacognitive ability have been associated with pathological behavior (Hoven et al., 2019), such as excessive checking behavior when having low confidence (Baptista, Maheu, Mallet, & N'Diaye, 2021).

Traditionally, theories have placed dysfunctions of metacognition at the center of obsessive–compulsive disorder (OCD) etiology (Purdon & Clark, 1999; Wells & Papageorgiou, 1998). Varying notions about the nature of these dysfunctions have been proposed. A first notion suggests that OCD patients suffer from a negative bias in confidence, resulting in underconfidence relative to healthy control subjects. This underconfidence may not necessarily be a defect in judging one's performance, since it could be an appropriate correction of the usual overconfidence seen in healthy individuals (Johnson & Fowler, 2011). Nevertheless, it could lead to excessive doubts, low self-beliefs and obsessive thoughts which could in turn promote compulsive behaviors, while checking behavior itself can also provoke feelings of low confidence (Jaeger et al., 2021; Radomsky, Gilchrist, & Dussault, 2006). Indeed, a recent meta-analysis of 19 studies covering a variety of cognitive tasks indicated that patients with OCD showed general underconfidence, in both cognitive domains of memory and perception (i.e. less confident than they should be considering their performance) (Dar, Sarna, Yardeni, & Lazarov, 2022). These studies focused mostly on local confidence judgments while doing specific tasks [i.e., trial by trial estimates on the correctness of a decision (Pouget, Drugowitsch, & Kepecs, 2016)] with the underlying assumption that underconfidence on a local level is related to clinically relevant subjective experiences of doubts such as decreased self-beliefs (i.e. higher order metacognition), but this has not yet been investigated. Recent studies suggest that local confidence and self-beliefs may be linked by more global estimates of confidence (e.g., confidence about performance on multiple decisions or a task) and that investigating the interplay between these hierarchical levels of confidence may bridge this gap (Seow, Rouault, Gillan, & Fleming, 2021).

A second notion suggests that perhaps not underconfidence, but an impairment in estimating or properly utilizing confidence judgments lies at the heart of OCD symptoms, particularly for compulsive behavior. This might manifest as a decreased sensitivity to identify correct from incorrect decisions using confidence judgments (i.e., decreased metacognitive sensitivity) (Hauser et al., 2017; Rouault, Seow, Gillan, & Fleming, 2018b) or a decoupling between levels of metacognition (Hoven, Luigjes, Denys, Rouault, & van Holst, 2023). As a result, patients might be less capable to self-correct and inform their future decisions using their confidence, and thus revert to compulsive behavior.

We will test these two notions using a behavioral protocol probing three hierarchical levels of confidence. The hypothesis put forward by the first notion is that relative underconfidence will be found in OCD patients at all three levels. The expectation that follows from the second notion is an impairment in using confidence judgements to separate correct from incorrect choices (i.e., metacognitive sensitivity). Note that these two notions are not mutually exclusive, and could simultaneously exist. However, following the second notion, a decoupling between different levels of metacognition could be expected which opposes the first notion of underconfidence across the three levels.

The relationship between obsessive–compulsive (OC) symptoms and metacognition has also been studied using general population samples, with the advantage of probing large samples with less time and costs investments, while also sampling larger symptom variability. Three such studies did not find evidence for a direct relationship between local confidence and OC symptoms (Benwell, Mohr, Wallberg, Kouadio, & Ince, 2022; Hoven et al., 2023; Rouault et al., 2018b), while another study did find a positive relationship, indicating that increased OC symptoms related to higher confidence (Seow & Gillan, 2020). Moreover, high OC symptoms in the general population have been related to decreases in metacognitive sensitivity, also without a difference in local confidence (Hauser et al., 2017). Overall, there is no evidence for decreased confidence, but some indication of reduced metacognitive sensitivity in these samples. The assumption of these types of studies is that there is a spectrum of OCD symptomatology where highly compulsive individuals resemble (albeit to a lesser extent) OCD patients in terms of possibly disturbed (meta)cognitive processes. However, the comparability of OCD patients and highly compulsive individuals has not been directly tested using carefully matched groups. Since clinical studies and general population studies have reported mixed findings regarding the relationship between OC symptoms and metacognition, these populations might be inherently different. In terms of metacognitive functioning, highly compulsive individuals from the general population could (1) resemble OCD patients (to a lesser extent) regarding both decreased confidence levels and metacognitive sensitivity, (2) only resemble OCD patients regarding decreased usage of confidence (i.e., decreased sensitivity, decreased coupling between metacognitive levels), or (3) be inherently different from OCD patients.

To test this, here we compared OCD patients not only to healthy subjects, but also to a group of matched highly compulsive individuals, on a wide range of metacognitive functions and their relationship with compulsive symptoms. We investigate both local confidence, global confidence, and higher-order self-beliefs to obtain an inclusive picture of metacognitive abilities in people suffering from OC symptoms. We expect (as preregistered: https://osf.io/3knjc) decreased local and global confidence in OCD patients compared to healthy controls (HCs), as well as decreased self-beliefs (i.e., self-esteem, autonomy). Moreover, since OCD patients were found to be more reliant on external feedback when assessing their confidence (Lazarov, Liberman, Hermesh, & Dar, 2014), we expected that underconfidence in OCD patients would be more pronounced in trials without feedback and with increased symptom severity. Also, we expect lower metacognitive sensitivity in OCD patients, resulting in a decreased ability to use local confidence to differentiate between correct and incorrect answers (i.e., discrimination), and we expect a distorted relationship between local and global confidence in OCD as well. Finally, we test whether abnormalities in metacognition found in OCD resemble those of matched highly compulsive individuals.

## Materials and methods

### Ethics

All experimental procedures were approved by the Medical Ethics Committee of the Amsterdam University Medical Centre. All participants provided written informed consent before the start of any experimental procedure and were reimbursed for their time.

### Participants

In this study we collected data from three groups: HCs, OCD patients and highly compulsive non-clinical subjects. We did not perform an a-priori power analysis for the sample sizes of these three groups. Instead, we based our sample size on similar studies assessing clinical populations [e.g. (Marton et al., 2019; Radomsky, Dugas, Alcolado, & Lavoie, 2014; Vaghi et al., 2017)].

### OCD patients

Forty-five patients with OCD, aged between 18 and 65 years old were included. They were recruited via various local treatment centers and patient associations across the Netherlands, and previously and/or currently underwent psychotherapy. The average duration of symptoms in the patient group was 19.3 years with an average time since diagnosis of 9.2 years. Severity as measured by the Y-BOCS (mean: 21.88 ± 5.84) indicated to be in the upper range of moderate and lower range of severe symptom strength. Exclusion criteria included diagnoses of any comorbid psychiatric disorders, and the use of any medication for the treatment of psychiatric symptoms, including, but not limited to, selective serotonin reuptake inhibitors, tricyclic antidepressants, or antipsychotics. After applying task-based exclusion criteria of lower than chance level performance or too little variation in confidence judgements (for more extensive description see (Hoven et al., 2023), our final sample consisted of 40 OCD patients.

### Healthy controls

Forty-five HCs were included in this study, between 18 and 65 years old. They were recruited through online advertisements and from our participant database across the Netherlands. HCs were matched to OCD patients on age, sex and education levels. Exclusion criteria included diagnoses of any psychiatric disorder or the use of any psychotropic medication. After applying task-based exclusion criteria (Hoven et al., 2023), our final sample consisted of 40 HCs.

### High-compulsive subjects

As part of a larger previous study, 625 English speaking world-wide participants were collected online via the Prolific Academic platform (www.prolific.co) (see Hoven et al., 2023 for more details). Subjects were not screened for psychiatric diagnoses, since our aim was to collect data based on continuous variation in psychiatric symptoms within the general population. We excluded subjects who failed attention and comprehension checks, and used the same task-based exclusion criteria as in the clinical sample, and the final sample consisted of 489 subjects. Then we performed propensity score matching in order to select subjects from our large general population sample (*N* = 489) to match our patient sample in terms of obsessive–compulsive symptoms. Using the MatchIt package in R (Ho, Imai, & Imai, 2013) we performed nearest neighbor matching. We matched our OCD patient sample to an equal number of highly compulsive subjects from the general population sample based on Obsessive–Compulsive Inventory Revised (OCI-R) score, age, sex and education level (Foa et al., 2002). Our final sample thus consisted of three sets of 40 subjects: 40 OCD patients, 40 HCs and 40 high-compulsive subjects (HComp) from the general population study. Demographics were compared between groups using two-sample *t* tests for continuous measures or Chi-square tests for categorical measures.

### Questionnaires

All HCs and OCD patients were subjected to the MINI structured psychiatric interview (Sheehan et al., 1998) to screen for any (co-morbid) psychiatric disorders. OCD symptom severity was measured using the Obsessive-Compulsive Inventory – Revised (OCI-R) (Foa et al., 2002). All our 120 subjects were assessed with questionnaires on autonomy (Autonomy Scale Amsterdam: ASA) (Bergamin et al., in prep) and self-esteem (Rosenberg Self-Esteem Scale: rSES) (Rosenberg, 1965) as measures of higher-order self-beliefs. Moreover, anxiety and depression symptoms were assessed using the Depression Anxiety and Stress Scale (DASS) (Parkitny & McAuley, 2010) in the clinical sample (OCD and HC) and using the Generalized Anxiety Disorder-7 questionnaire (Williams, 2014), and Zung's depression scale (Zung, 1965), respectively, in the general population (HComp) sample. Metacognitive beliefs were measured in the clinical sample using the Metacognitions Questionnaire-30 (MCQ-30) (Wells & Cartwright-Hatton, 2004).

### Local and global confidence task

The perceptual decision-making task was adapted from *Experiment 3* in Rouault et al. (2019) and was coded in JavaScript, HTML and CSS using jsPsych version 4.3 and hosted on Gorilla (gorilla.sc) (Anwyl-Irvine, Massonnié, Flitton, Kirkham, & Evershed, 2020). All subjects performed the task online using their personal computer.

**Figure 1.**
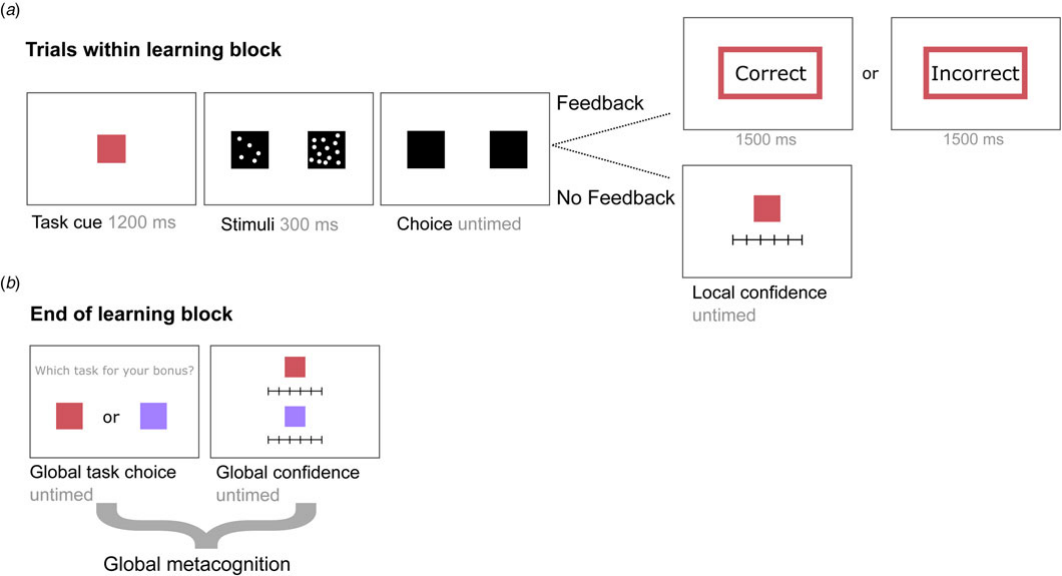
Experimental design. (*a*) Participants performed learning blocks with two randomly alternating trials from two tasks, indicated by a task cue. Each task was either easy or difficult and provided feedback or no feedback (2 × 2 design), resulting in six different possible task pairings. Each trial started with the presentation of a color cue, indicating which of the two tasks was presented, after which subjects had to choose which of two boxes contained a higher number of dots. Each judgment was either easy or difficult, dependent on the dot difference between the boxes. After their choice, subjects either received feedback (correct or incorrect) about their choice, or did not receive feedback and instead were asked to provide a local confidence rating about the probability of their perceptual judgment being correct. (*b*) At the end of each learning block participants were asked to choose which task should be used to calculate a bonus based on their performance; global task choice. They also rated their overall ability; global confidence. Both are measures of global metacognition.

All participants performed blocks with two randomly interleaved perceptual tasks (with six pseudo-randomized trials each) indicated by two color cues (Fig. 1). Participants had to indicate which of two black boxes contained a higher number of white dots. Two experimental features were implemented: a task could be easy or difficult (i.e., difficulty feature), and could deliver veridical feedback or no feedback (i.e., feedback feature), resulting in six possible pairings of tasks within each block. All six possible pairings occurred twice in randomized order, resulting in 144 trials per participant. On each trial without feedback (72 trials per participant) participants indicated their *local confidence* about their probability of being correct on that specific trial on a scale from ‘50% correct (chance level)’ to ‘100% correct (perfect)’. At the end of each block participants had to indicate the task in which they believed they performed best. Moreover, participants rated their confidence in their overall performance on each of the two tasks (*global confidence*) on a scale from 50% to 100%. For more detailed information on the task specifics, see Hoven et al. (2023).

### Task-based measures of metacognition

Using local and global confidence, we calculated *local calibration* (decision level), which is the difference between average local confidence and performance on no-feedback tasks only. *Global calibration* (task level) was calculated as the difference between average global confidence and performance on all trials. These measures reflect how well one's confidence matches one's actual performance and can be interpreted as overconfidence (when positive) or underconfidence (when negative). We also calculated the direct correlation between average local and global confidence per subject on no-feedback tasks only. Note that for one OCD patient this correlation could not be determined due to a lack of variance in their global confidence. Moreover, we computed *discrimination*, which is a metric of metacognitive sensitivity that indicates how well one's confidence judgments discriminate between their own correct and incorrect choices. It is calculated as the difference between the average confidence for correct and the average confidence for incorrect trials. Another metric to assess metacognitive sensitivity is meta-d’ (Fleming, 2017), whose computations are known to be imprecise in designs with a low number of trials per subject per condition (Rouault, McWilliams, Allen, & Fleming, 2018a) (in our case, 36 trials). Moreover, since results from earlier work (Lebreton et al., 2018) showed high correlations between discrimination and meta-d’, we used the discrimination metric as our measure of metacognitive sensitivity in the current study.

### Analyses

All analyses were performed using RStudio (version 2022.07.2). Mixed ANOVAs (afex package in R (Singmann, Bolker, & Westfall, 2015)) were used to investigate the effects of group, difficulty and feedback on: accuracy, reaction times, global task choice and global confidence, and to investigate the effects of group and difficulty on local confidence. Using this approach, we investigated whether OCD patients showed metacognitive deviations compared to HCs, and importantly, whether metacognitive findings from a general population sample of HComp individuals are comparable to a clinical sample of OCD patients.

Two-sample *t* tests were used to compare local calibration, global calibration, discrimination, the correlation of local and global confidence, autonomy and self-esteem between (1) OCD and HC, and (2) OCD and HComp subjects. One sample *t* tests against 0 were performed to formally assess the existence over- or underconfidence for both local and global calibration in each of the three groups. Additionally, regression analyses were performed to explore differences between groups in how internal fluctuations in local confidence would predict global confidence, over and above fluctuations in accuracy or reaction times. For these regressions, only blocks without feedback were used (since only these blocks contained local confidence judgments). All predictors were standardized (z-scored). In this analysis we aimed to predict differences in global confidence between tasks using main effects and the interactions between group and the difference in accuracy, RT and local confidence between those tasks, as follows:
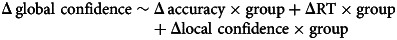


For all analyses where the measure of local confidence was used (i.e., local calibration, discrimination, correlation of local and global confidence), only the 72 trials from the no-feedback condition were used, since participants only rated their local confidence in those trials. In order to assess if there were differences in the relationship between obsessive–compulsive symptom strength (OCI-R score) and metacognitive abilities between OCD patients and HComp subjects, we performed linear regressions on our metacognition variables with OCI-R score, group and their interaction as predictors.

All analyses codes and anonymized data that will reproduce the figures can be found at https://osf.io/ksfp6/.

## Results

### Demographics

**Figure 2.**
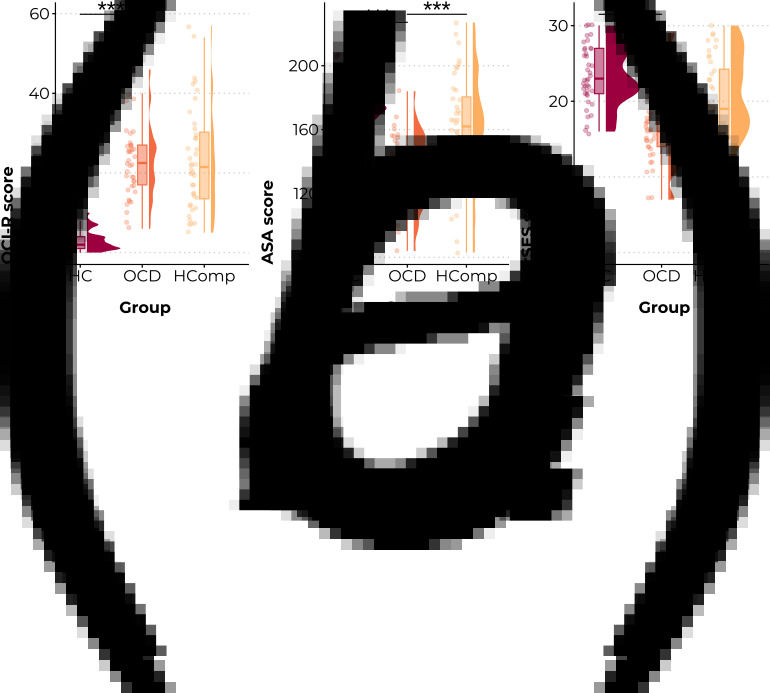
Clinical scores across groups. Scores on the (*a*) OCI-R score, (*b*) ASA score reflecting autonomy and (*c*) rSES score reflecting self-esteem per group. Dots show data from individual participants, boxplots show median and upper/lower quartile with whiskers indicating the 1.5 interquartile range, distributions show the probability density function of all data points per group. *p < 0.05, **p < 0.01, ***p < 0.001. HC, healthy control subjects; OCD, obsessive–compulsive disorder patients; HComp, highly compulsive subjects from general population sample; OCI-R, Obsessive–Compulsive Inventory-Revised; ASA, Autonomy Scale Amsterdam; rSES, Rosenberg's Self Esteem Scale.

Demographic and clinical characteristics are given in Table 1. The groups did not differ in terms of age, sex distribution or years of education. OCD patients have significantly higher OCI-R scores than HCs, while OCI-R scores were similar between OCD patients and HComp subjects (Fig. 2*a*). Together, this confirms successful matching of the groups. For details on all descriptive statistics and statistical outcomes, see Table 1. For correlations between questionnaires, see online Supplementary Table S1.

**Table 1.** Demographics, clinical data and task performance per group and differences between groups

	OCD (*N* = 40)	HCs (*N* = 40)	HComp (*N* = 40)	OCD *v.* HCs	OCD *v.* HComp
Demographics					
Age in years	38.18 (11.22)	38.58 (11.11)	36.53 (12.73)	*T* = 0.16 *p* = 0.87	*T* = 0.61 *p* = 0.54
Females (%)	26 (65%)	27 (67.5%)	28 (70%)	χ^2^ = 0.81 *p* = 0.81	χ^2^ = 0.23 *p* = 0.63
Years of education	10.11 (3.21)	10.20 (3.13)	10.35 (2.64)	*T* = 0.12 *p* = 0.90	*T* = −0.36 *p* = 0.72
Questionnaire scores					
OCI-R	23.23 (9.43)	2.90 (2.48)	23.35 (13.18)	*T* = −13.19 *p* < 0.001	*T* = −0.05 *p* = 0.96
ASA	133.33 (21.70)	168.13 (19.18)	160.35 (33.99)	*T* = −7.60 *p* < 0.001	*T* = 4.24 *p* < 0.001
rSES	16.95 (4.89)	23.48 (3.94)	18.53 (7.56)	*T* = −6.57 *p* < 0.001	*T* = 1.11 *p* = 0.273
DASS	34.2 (17.31)	4.98 (4.23)		*T* = −10.37 *p* < 0.001	
DASS anx	9.05 (6.59)	0.68 (0.92)		*T* = −7.96 *p* < 0.001	
DASS dep	10.38 (8.28)	1.28 (1.47)		*T* = −6.84 *p* < 0.001	
MCQ-30	66.10 (15.45)	42.88 (8.79)		*T* = −8.26 *p* < 0.001	
GAD-7			9.08 (6.20)		
ZungDEP			44.78 (11.11)		
Metacognition					
Accuracy (percent correct)	75.04 (7.00)	76.49 (7.76)	69.90 (8.64)	*F* = 0.81 *p* = 0.372 *η*^2^_G_ = 0.006	*F* = 8.59 *p* = 0.004 *η*^2^_G_ = 0.061
Local confidence (on 50–100 scale)	74.74 (8.11)	81.14 (8.04)	76.82 (9.58)	*F* = 12.59 *p* < 0.001 *η*^2^_G_ = 0.129	*F* = 1.11 *p* = 0.296 *η*^2^_G_ = 0.013
Global confidence	76.24 (7.27)	80.69 (7.34)	76.21 (8.83)	*F* = 7.42 *p* = 0.008 *η*^2^_G_ = 0.069	*F* = 0.0002 *p* = 0.989 *η*^2^_G_ = 2.1.10^−6^
Local calibration	-0.17 (11.83)	4.82 (8.92)	6.63 (11.22)	*T* = 2.13 *p* = 0.036 *d* = 0.48	*T* = 2.64 *p* = 0.010 *d* = 0.59
Global calibration	1.20 (8.84)	4.20 (6.98)	6.31 (9.62)	*T* = 1.68 *p* = 0.096 *d* = 0.38	*T* = 2.48 *p* = 0.015 *d* = 0.55
Correlation local & global confidence	0.51	0.56	0.52	*T* = −0.80 *p* = 0.429 *d* = 0.18	*T* = 0.18 *p* = 0.862 *d* = 0.04
Discrimination	9.40 (5.94)	8.34 (4.77)	6.73 (4.66)	*T* = 0.88 *p* = 0.383 *d* = 0.20	*T* = −2.24 *p* = 0.028 *d* = 0.50

OCD, obsessive–compulsive disorder; HCs, healthy controls; HComp, highly compulsive subjects; OCI-R, Obsessive-Compulsive Inventory-Revised; ASA, Autonomy Scale Amsterdam; rSES, Rosenberg Self-Esteem Scale; DASS, Depression Anxiety and Stress Scale; DASS anx, Depression Anxiety and Stress – subscale Anxiety; DASS dep, Depression Anxiety and Stress – subscale Depression; GAD-7, Generalized Anxiety Disorder-7 Questionnaire; ZungDEP, Zung's Depression scale; T, T-value from two-sample *t* test; *F, F*-value from ANOVA; *P, p*-value, *η*^2^_G_, Generalized Eta-squared; d, Cohen's d.

Data are reported as mean (standard deviation).

### Replication analyses on task structure

Using mixed ANOVAs in our clinical sample, we replicated earlier findings investigating the effects of feedback and difficulty on performance and metacognition (Rouault et al., 2019). For performance, reaction times and global confidence we assessed the effects of feedback, difficulty and group, whereas for local confidence we assessed the effects of difficulty, accuracy and group. For none of the analyses interactions between task features and group were found.

In line with previous findings, performance was better for easy *v.* hard tasks [*F*(1,78) = 501.93, *p* < 0.001], but did not differ between feedback or no feedback conditions [*F*(1,78) = 0.14, *p* = 0.705]. Reaction times were faster for easy *v.* hard tasks [*F*(1,78) = 42.01, *p* < 0.001] and tasks that provided feedback *v.* no feedback [*F*(1,78) = 28.45, *p* < 0.001].

Global confidence was higher for easy *v.* hard tasks [*F*(1,78) = 87.58, *p* < 0.001], and for tasks providing feedback *v.* no feedback [*F*(1,78) = 101.92, *p* < 0.001], even though performance was equal between presence and absence of feedback. The difference in global confidence between feedback and no-feedback tasks was bigger when the tasks were easy [*F*(1,78) = 5.10, *p* = 0.0267]. As expected, local confidence was higher for easy *v.* hard tasks [*F*(1,78) = 114.99, *p* < 0.001], and for correct *v.* incorrect trials [*F*(1,78) = 217.01, *p* < 0.001]. Together, these results largely confirm previous observations on this protocol (Hoven et al., 2023; Rouault et al., 2019).

### Comparing OCD patients to healthy controls

**Figure 3.**
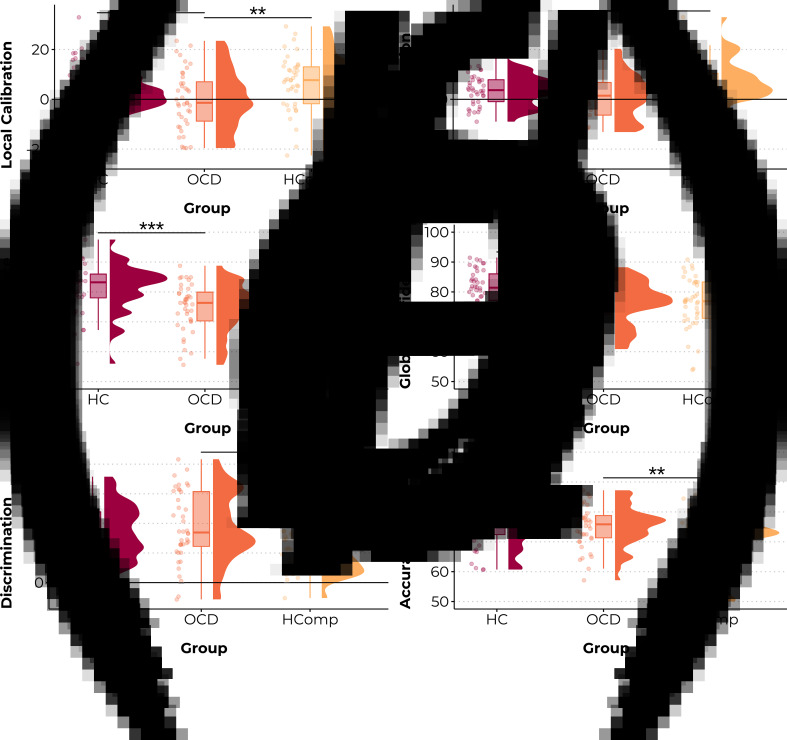
Metacognition and performance across groups. Local calibration (*a*), global calibration (*b*), local confidence (*c*), global confidence (*d*), discrimination (*e*), and accuracy (*f*) data, all in percentages. Dots show data from individual participants, boxplots show median and upper/lower quartile with whiskers indicating the 1.5 interquartile range, distributions show the probability density function of all data points per group. For plots A, B and E significance stars represent two-sample *t* tests, for plots C, D and F significance stars represent the main effect of group in mixed ANOVAs (see Table 1). *p < 0.05, **p < 0.01, ***p < 0.001. HC, healthy control subjects; OCD, obsessive–compulsive disorder patients; HComp, highly compulsive subjects from the general population sample.

In line with our expectations, OCD patients showed significantly lower local calibration compared with HCs, and a trend level of lower global calibration, indicating underconfidence relative to HCs (Table 1, Fig. 3*a*, *b*). These results were due to significantly decreased local and global confidence levels in OCD compared with HCs, without any performance or reaction time differences (Fig. 3*c*, *d*, *f*). One sample *t* tests against zero indicated that HCs showed significant local (*t*_39_ = 3.42, *p* = 0.001) and global overconfidence (*t*_39_ = 3.81, *p* < 0.001), while local and global calibration did not differ from zero in the OCD group, indicating that the OCD group was well calibrated (local: *t*_39_ = −0.09, *p* = 0.928, global: *t*_39_ = 0.86, *p* = 0.397). Moreover, autonomy (as measured by the ASA), self-esteem (as measured by the rSES) were found to be significantly lower in patients with OCD compared with HCs (Fig. 2*b*, *c*), while metacognitive beliefs (as measured by the MCQ-30) were significantly more distorted in OCD (Table 1).

No significant interactions between task parameters (feedback or difficulty) and group were found, refuting our hypothesis that OCD patients would especially show lower global confidence when feedback was unavailable. Also, no group differences in discrimination or the correlation between local and global confidence were found (Table 1, Fig. 3*e*).

It has been argued that the findings of decreased confidence in OCD in case–control studies could be driven by comorbid depressive and anxiety symptoms, while compulsivity would contrarily lead to increased (over)confidence (Rouault et al., 2018b; Seow & Gillan, 2020). We performed regression analyses investigating the effect of group (OCD *v.* HC) on local and global confidence and calibration, while controlling for anxiety and depression symptoms (DASS scores). The effect of group on all four metacognitive outcome measures remained significant (local confidence: *β* = −8.508 ± 2.785, *p* = 0.003; global confidence: *β* = −6.027 ± 2.526, *p* = 0.0195; local calibration: *β* = −11.091 ± 3.521, *p* = 0.002; global calibration: *β* = −7.234 ± 2.691, *p* = 0.009; see online Supplementary Table S3 for full regression results). This suggests that in this clinical case–control sample decreases in confidence in OCD compared to HCs were not explained away by comorbid anxiety and depression symptoms.

### Comparing OCD patients to highly compulsive subjects

HComp subjects had significantly higher calibration (i.e. more overconfidence) at both local and global levels compared to OCD patients (Table 1, Fig. 3*a*, *b*). One sample *t* tests against zero confirmed that the HComp group showed significant local (*t*_39_ = 3.73, *p* < 0.001) and global overconfidence (*t*_39_ = 4.15, *p* < 0.001). This was due to a significantly worse performance of HComp subjects compared with OCD patients, while local and global confidence levels (and reaction times) did not differ between groups (Fig. 3*c*, *d*, *f*). In other words, HComp subjects were just as confident in their decisions as OCD patients, while performing significantly worse, leading to overconfidence. Moreover, autonomy was significantly lower in patients with OCD compared with HComp subjects, but there were no group differences in self-esteem scores (Fig. 2*b*, *c*).

HComp subjects showed decreased discrimination compared with OCD patients, indicating that the difference in confidence between correct and incorrect choices was smaller in this group, reflecting worse metacognitive sensitivity (Fig. 2*e*). However, no group differences were found in the correlation between local and global confidence. Again, we did not find any significant interaction effects between task parameters (feedback or difficulty) and group.

**Figure 4.**
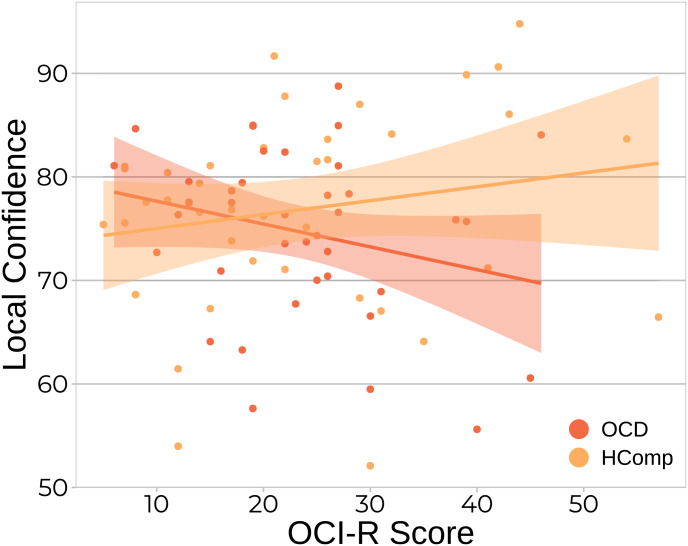
The relationship between local confidence and OCI-R scores in OCD patients and highly compulsive non-clinical subjects. Individual data points showing the relationship between OCI-R score and local confidence, which is negative in the OCD group, and positive in the HComp group. OCD, obsessive–compulsive disorder patients; HComp, highly compulsive subjects from the general population sample.

To deepen our understanding of the relationships between obsessive-compulsive symptoms and metacognition beyond group differences, we investigated if OCD patients and HComp subjects showed a different relationship between obsessive-compulsive symptom strength and metacognitive ability. Using regression analyses, a trend level interaction effect of OCI-R score and group on local confidence was found (*β* = 4.03 ± 2.09, *p* = 0.057, see online Supplementary Table S4 for full regression results). This interaction effect hints at a negative relationship in the OCD patients (i.e. more symptoms reflect lower local confidence), and a positive relationship in the HComp group (i.e. more symptoms reflect higher local confidence), however, post-hoc correlational tests did not show significance for the groups separately (OCD: *r* = −0.26, *t*_38_ = −1.63, *p* = 0.11; HComp: *r* = 0.18, *t*_38_ = 1.16, *p* = 0.25) (Fig. 4).

### Comparing healthy controls to highly compulsive subjects

For completeness, we performed exploratory analyses to compare the HC and HComp groups using the same methods as were used to compare the other groups. For results, see online Supplementary Materials (online Supplementary Table S2).

### Interplay between hierarchical levels of metacognition

Using regression analyses we replicated in our clinical sample that differences in local confidence between two tasks significantly inform global confidence differences between those tasks (*β* = 6.57 ± 1.21, *p* < 0.001), over and above differences in objective accuracy (*β* = −0.32 ± 1.05, *p* = 0.761) or reaction times (*β* = 0.22 ± 1.05, *p* = 0.831). No interaction effects with group were found, suggesting that the relationship between local and global confidence did not differ between OCD patients and HCs, or between OCD patients and HComp subjects. This is in line with non-significant group differences between the correlation coefficients of local and global confidence (Table 1).

## Discussion

Human research in psychiatry has historically been carried out by examining either clinical patient samples or psychiatric symptoms at subclinical or clinical levels in samples from the general population. It is assumed, but hardly ever formally tested, that psychological or cognitive processes that play a role in the symptoms in question are comparable between clinical patient samples and general population samples (Abramowitz et al., 2014). The current study tested this assumption by directly comparing carefully matched clinical and analog groups on cognitive processes central to the development and maintenance of OCD.

In line with our hypotheses and the notion of a negative confidence bias (Dar et al., 2022), the current study shows decreased local confidence in patients with OCD compared to HCs, with no performance differences, where HC are overconfident and OCD patients are relatively more underconfident. Interestingly, this negative bias extended to higher-order levels of metacognition, both task-based and questionnaire-based. Patients with OCD compared to HCs had decreases in global confidence, self-esteem and autonomy, and more distorted metacognitive beliefs. However, critically, OCD patients showed no impairments in confidence estimation or usage: they were just as good in discriminating between correct and incorrect choices using their confidence judgments (i.e., measured using discrimination), did not show specifically decreased confidence in trials without feedback, and showed no distortion of the relationship between local and global confidence. Overall, this supports the notion of a general negative bias across hierarchical metacognitive levels, reflecting the wide-spread nature of these deficits in OCD, with no evidence for disturbances in the estimation and usage of confidence. It remains possible, however, that deficits in metacognitive sensitivity and coupling of metacognitive levels would be more pronounced in clinically relevant contexts than in the current neutral perceptual task (Hoven et al., 2019).

Interestingly, the metacognitive pattern of the highly compulsive general population sample was different from the OCD sample, challenging the assumption that these two sample types are directly comparable. Contrary to the notion of a negative confidence bias in OCD samples, HComp subjects were significantly more overconfident – both at the local (decision) and global (task) levels – than patients with OCD, which was driven by decreased performance with equal confidence. Importantly, the metacognitive aberrancies of HComp did not resemble those of OCD patients. Instead, they were in the opposite direction: HComp individuals had relatively higher overconfidence (albeit not significant, see Supplementary Materials) than HCs. Moreover, directly going against the assumption of similar associations between symptoms and cognitive processes for clinical and general population samples, there were tentative opposite associations between OC symptoms and local confidence in patients with OCD (negative relationship) and HComp subjects (positive relationship). In line with previous findings of a decreased metacognitive sensitivity (Hauser et al., 2017), HComp subjects were worse in discriminating errors from correct answers using their confidence judgments compared with OCD patients.

Unlike most prior case–control studies in OCD, here we controlled for the influence of comorbid symptomatology (e.g. anxiety and depression) on confidence in patients with OCD. Since depression is associated with decreases in confidence (Hoven et al., 2019), it could partly explain lower confidence in OCD. We found, however, that decreases in local and global confidence and calibration levels in OCD compared to HCs remained when controlling for anxiety and depression symptoms. Additionally, anxiety and depression scores in OCD and HComp groups (using the DASS in OCD, and GAD-7 and Zung Depression Scale in HComp) both indicated mild severity. It is thus unlikely that the opposite metacognitive patterns we found are due to strong differences in comorbid symptoms between these samples. In the same line, a possible explanation is that decreased calibration (i.e. relative underconfidence) as found in our OCD sample relates more strongly to (anxiety driven) obsessive symptoms, whereas overconfidence or defects in metacognitive sensitivity would relate more strongly to compulsive symptoms. Yet, obsessive and compulsive symptoms, as measured by the Y-BOCS in the patients, were on average equally severe, going against the idea that more severe obsessions *v.* compulsions would drive underconfidence.

To account for comorbidities and heterogeneity within OCD and other disorders, a case has been made for transdiagnostic, dimensional approaches (Insel et al., 2010). Studies with large general population samples found that a symptom cluster of ‘Compulsive Behavior and Intrusive Thoughts’ (CIT), mostly including symptoms of OCD, schizotypy, eating disorders, alcoholism and impulsivity, was related to increases in local confidence, whereas a symptom cluster of ‘Anxious Depression’ (AD) was related to decreases in local confidence, while disorder-specific symptoms did not show these associations (Benwell et al., 2022; Rouault et al., 2018b; Seow & Gillan, 2020). In recent work, we extended these findings showing that CIT symptoms related to local and global overconfidence, while AD symptoms related to local and global underconfidence (Hoven et al., 2023). In light of previous findings that AD symptoms lead to lower confidence, while CIT symptoms lead to higher confidence, it could be that our current general population sample has higher CIT symptom dimension scores than the OCD sample which may additionally include non-OCD symptoms. Moreover, in the OCD sample we found lower confidence even when corrected for anxiety and depression symptoms. This questions the idea that the symptom dimensions and their relation with confidence biases may directly translate to a clinical population, at least in the case of OCD and compulsive symptoms. Although caution is warranted in generalizing transdiagnostic findings to clinical populations, transdiagnostic research is valuable in itself (McGorry, Hartmann, Spooner, & Nelson, 2018; Vanes & Dolan, 2021). An impactful step forward would be to apply transdiagnostic research within clinical samples. Recently, within a large patient sample of generalized anxiety disorder and OCD patients, it was found that deficits in goal-directed behavior were more strongly associated with a dimension of compulsivity symptoms than OCD diagnosis status itself (Gillan et al., 2020), supporting the importance of studying both transdiagnostic symptoms and diagnostic criteria in concert in clinical samples.

The current study has to be interpreted in light of its limitations. Because of the difficulty manipulation in the experimental design, we did not use a staircase procedure, and used calibration measures to analyze the strength of correspondence between confidence and performance. Differences in performance between the OCD and HComp group were found, with a negative relationship between OCI-R score and performance in the large general population sample (Hoven et al., 2023). Including subjects' mean performance in the propensity score matching strongly worsened the matching on our primary variable of interest, the OCI-R score, which is why we did not pursue matching on performance. In next studies it would be useful to keep performance equal between participants to more clearly isolate changes in confidence. Our clinical sample consisted of Dutch OCD patients that were help-seeking, did not use psychotropic medication at time of testing and did not suffer from co-morbid diagnoses. This allowed us to isolate associations with metacognition without these confounds, but could limit the generalizability of our findings to the general OCD patient population, because co-morbidities and medication use are common in OCD (Grabe et al., 2000; Ruscio, Stein, Chiu, & Kessler, 2008). Moreover, all subjects were tested online (and originated from a variety of countries), allowing for less control over the environment in which the task was performed. Nevertheless, online testing has many advantages, including lower costs and access to larger and more representative samples. Future studies could investigate metacognition in a more clinically relevant setting, by – for example – studying the effects of symptom provocation on metacognitive abilities, and could study the specific role of obsessions v. compulsions in metacognition. Moreover, metacognition does not only serve monitoring purposes, but also has a controlling function, which should be investigated further in OCD (Vaghi et al., 2017).

Together, these findings argue for being cautious in generalizing metacognitive findings from highly compulsive samples from the general population to clinical samples. In our current samples, with equal OC symptom severity, distinct neurocognitive processes might be at play, relating to OC symptoms in different ways. This caution might not apply similarly to all psychiatric disorders, since for example, both clinical and general population studies have consistently shown decreases in confidence in depression (Hoven et al., 2019; Rouault et al., 2018b). Overall, the current study showed evidence for decreased local and global confidence, as well as decreased higher order metacognition in OCD patients compared with HCs. Meanwhile, a general population sample with similar OC symptoms showed local and global overconfidence and diminished metacognitive sensitivity compared with OCD patients. The patterns observed in a non-clinical population, used as an analog for OCD, may thus not necessarily generalize to clinical samples.

## Supporting information

Hoven et al. supplementary material Hoven et al. supplementary material

## Supplementary material

The supplementary material for this article can be found at https://doi.org/10.1017/S003329172300209X.

## References

[ref1] Abramowitz, J. S., Fabricant, L. E., Taylor, S., Deacon, B. J., McKay, D., & Storch, E. A. (2014). The relevance of analogue studies for understanding obsessions and compulsions. Clinical Psychology Review, 34(3), 206–217. 10.1016/j.cpr.2014.01.004. 24561743

[ref2] Anwyl-Irvine, A. L., Massonnié, J., Flitton, A., Kirkham, N., & Evershed, J. K. (2020). Gorilla in our midst: An online behavioral experiment builder. Behavior Research Methods, 52(1), 388–407. 10.3758/S13428-019-01237-X/TABLES/8. 31016684 PMC7005094

[ref3] Balsdon, T., Wyart, V., & Mamassian, P. (2020). Confidence controls perceptual evidence accumulation. Nature Communications, 11(1), 1–11. 10.1038/s41467-020-15561-w. PMC7145794 32273500

[ref4] Baptista, A., Maheu, M., Mallet, L., & N'Diaye, K. (2021). Joint contributions of metacognition and self-beliefs to uncertainty-guided checking behavior. Scientific Reports, 11(1), 1–10. 10.1038/s41598-021-97958-1. 34561475 PMC8463683

[ref5] Benwell, C. S. Y., Mohr, G., Wallberg, J., Kouadio, A., & Ince, R. A. A. (2022). Psychiatrically relevant signatures of domain-general decision-making and metacognition in the general population. NPJ Mental Health Research, 1(1), 1–17. 10.1038/s44184-022-00009-4. PMC10956036 38609460

[ref6] Cortese, A. (2022). Metacognitive resources for adaptive learning. Neuroscience Research, 178(March 2021), 10–19. 10.1016/j.neures.2021.09.003. 34534617

[ref7] Dar, R., Sarna, N., Yardeni, G., & Lazarov, A. (2022). Are people with obsessive–compulsive disorder under-confident in their memory and perception? A review and meta-analysis. Psychological Medicine, 52, 1–9. 10.1017/S0033291722001908. PMC9647546 35848286

[ref8] Desender, K., Murphy, P., Boldt, A., Verguts, T., & Yeung, N. (2019). A postdecisional neural marker of confidence predicts information-seeking in decision-making. Journal of Neuroscience, 39(17), 3309–3319. 10.1523/JNEUROSCI.2620-18.2019. 30804091 PMC6788827

[ref9] Fleming, S. M. (2017). HMeta-d: Hierarchical Bayesian estimation of metacognitive efficiency from confidence ratings. Neuroscience of Consciousness, 2017(1), 1–14. 10.1093/nc/nix007. PMC5858026 29877507

[ref10] Fleming, S. M., Dolan, R. J., & Frith, C. D. (2012). Metacognition: Computation, biology and function. Philosophical Transactions of the Royal Society B: Biological Sciences, 367(1594), 1280–1286. 10.1098/rstb.2012.0021. PMC3318771 22492746

[ref11] Foa, E. B., Huppert, J. D., Leiberg, S., Langner, R., Kichic, R., Hajcak, G., & Salkovskis, P. M. (2002). The obsessive–compulsive inventory: Development and validation of a short version. Psychological Assessment, 14(4), 485–496. 10.1037/1040-3590.14.4.485. 12501574

[ref12] Gillan, C. M., Kalanthroff, E., Evans, M., Weingarden, H. M., Jacoby, R. J., Gershkovich, M., … Simpson, H. B. (2020). Comparison of the association between goal-directed planning and self-reported compulsivity vs obsessive-compulsive disorder diagnosis. JAMA Psychiatry, 77(1), 77–85. 10.1001/jamapsychiatry.2019.2998. 31596434 PMC6802255

[ref13] Grabe, H. J., Meyer, C., Hapke, U., Rumpf, H. J., Freyberger, H. J., Dilling, H., & John, U. (2000). Prevalence, quality of life and psychosocial function in obsessive–compulsive disorder and subclinical obsessive–compulsive disorder in northern Germany. European Archives of Psychiatry and Clinical Neuroscience, 250(5), 262–268. 10.1007/s004060070017. 11097170

[ref14] Guggenmos, M., Wilbertz, G., Hebart, M. N., & Sterzer, P. (2016). Mesolimbic confidence signals guide perceptual learning in the absence of external feedback. ELife, 5, 1–19. 10.7554/eLife.13388. PMC4821804 27021283

[ref15] Hauser, T. U., Allen, M., Rees, G., Dolan, R. J., Bullmore, E. T., Goodyer, I., … Pantaleone, S. (2017). Metacognitive impairments extend perceptual decision making weaknesses in compulsivity. Scientific Reports, 7(1), 1–10. 10.1038/s41598-017-06116-z. 28747627 PMC5529539

[ref16] Ho, D., Imai, K., & Imai, M. K. (2013). Package ‘MatchIt.’. RStudio, 23, 2014. 10.1093/pan/mpl013>.Maintainer.

[ref17] Hoven, M., Lebreton, M., Engelmann, J. B., Denys, D., Luigjes, J., & van Holst, R. J. (2019). Abnormalities of confidence in psychiatry: An overview and future perspectives. Translational Psychiatry, 9(1), 1–18. 10.1038/s41398-019-0602-7. 31636252 PMC6803712

[ref18] Hoven, M., Luigjes, J., Denys, D., Rouault, M., & van Holst, R. J. (2023). How do confidence and self-beliefs relate in psychopathology: a transdiagnostic approach. Nature Mental Health, 1, 1–9.

[ref19] Insel, T., Cuthbert, B., Garvey, M., Heinssen, R., Pine, D. S., Quinn, K., … Wang, P. (2010). Research domain criteria (RDoC): Toward a new classification framework for research on mental disorders. American Journal of Psychiatry, 167(7), 748–751. 10.1176/appi.ajp.2010.09091379. 20595427

[ref20] Jaeger, T., Moulding, R., Yang, Y. H., David, J., Knight, T., & Norberg, M. M. (2021). A systematic review of obsessive–compulsive disorder and self: Self-esteem, feared self, self-ambivalence, egodystonicity, early maladaptive schemas, and self concealment. Journal of Obsessive–Compulsive and Related Disorders, 31, 100665. 10.1016/J.JOCRD.2021.100665.

[ref21] Johnson, D. D. P., & Fowler, J. H. (2011). The evolution of overconfidence. Nature, 477(7364), 317–320. 10.1038/nature10384. 21921915

[ref22] Lazarov, A., Liberman, N., Hermesh, H., & Dar, R. (2014). Seeking proxies for internal states in obsessive–compulsive disorder. Journal of Abnormal Psychology, 123(4), 695–704. 10.1037/abn0000004. 25133987

[ref23] Lebreton, M., Langdon, S., Slieker, M. J., Nooitgedacht, J. S., Goudriaan, A. E., Denys, D., … Luigjes, J. (2018). Two sides of the same coin: Monetary incentives concurrently improve and bias confidence judgments. Science Advances, 4(5), eaaq0668. 10.1126/sciadv.aaq0668. 29854944 PMC5976269

[ref24] Marton, T., Samuels, J., Nestadt, P., Krasnow, J., Wang, Y., Shuler, M., … Nestadt, G. (2019). Validating a dimension of doubt in decisionmaking: A proposed endophenotype for obsessive–compulsive disorder. PLoS ONE, 14(6), 1–14. 10.1371/journal.pone.0218182. PMC6564001 31194808

[ref25] McGorry, P. D., Hartmann, J. A., Spooner, R., & Nelson, B. (2018). Beyond the “at risk mental state” concept: Transitioning to transdiagnostic psychiatry. World Psychiatry, 17(2), 133–142. 10.1002/wps.20514. 29856558 PMC5980504

[ref26] Parkitny, L., & McAuley, J. (2010). The depression anxiety stress scale (DASS). Journal of Physiotherapy, 56(2), 204. 10.1016/s1836-9553(10)70030-8. 20795931

[ref27] Pescetelli, N., Hauperich, A. K., & Yeung, N. (2021). Confidence, advice seeking and changes of mind in decision making. Cognition, 215, 1–9. 10.1016/j.cognition.2021.104810. 34147712

[ref28] Pouget, A., Drugowitsch, J., & Kepecs, A. (2016). Confidence and certainty: Distinct probabilistic quantities for different goals. Nature Neuroscience, 19(3), 366–374. 10.1038/nn.4240. 26906503 PMC5378479

[ref29] Purdon, C., & Clark, D. A. (1999). Metacognition and obsessions. Clinical Psychology and Psychotherapy, 6(2), 102–110. 10.1002/(sici)1099-0879(199905)6:2<102::aid-cpp191>3.0.co;2-5.

[ref30] Radomsky, A. S., Dugas, M. J., Alcolado, G. M., & Lavoie, S. L. (2014). When more is less: Doubt, repetition, memory, metamemory, and compulsive checking in OCD. Behaviour Research and Therapy, 59, 30–39. 10.1016/j.brat.2014.05.008. 24952303

[ref31] Radomsky, A. S., Gilchrist, P. T., & Dussault, D. (2006). Repeated checking really does cause memory distrust. Behaviour Research and Therapy, 44(2), 305–316. 10.1016/j.brat.2005.02.005. 15890313

[ref32] Rollwage, M., Loosen, A., Hauser, T. U., Moran, R., Dolan, R. J., & Fleming, S. M. (2020). Confidence drives a neural confirmation bias. Nature Communications, 11(1), 1–11. 10.1038/s41467-020-16278-6. PMC7250867 32457308

[ref33] Rosenberg, M. (1965). Rosenberg self-esteem scale (RSE). Acceptance and Commitment Therapy. Measures Package., 61(52), 1–18. 10.32388/bcazmm.

[ref34] Rouault, M., Dayan, P., & Fleming, S. M. (2019). Forming global estimates of self-performance from local confidence. Nature Communications, 10(1), 1141. 10.1038/s41467-019-09075-3. PMC6408496 30850612

[ref35] Rouault, M., McWilliams, A., Allen, M. G., & Fleming, S. M. (2018a). Human metacognition across domains: Insights from individual differences and neuroimaging. Personality Neuroscience, 1, 1–13. 10.1017/pen.2018.16. PMC6217996 30411087

[ref36] Rouault, M., Seow, T., Gillan, C. M., & Fleming, S. M. (2018b). Psychiatric symptom dimensions are associated with dissociable shifts in metacognition but not task performance. Biological Psychiatry, 84(6), 443–451. 10.1016/j.biopsych.2017.12.017. 29458997 PMC6117452

[ref37] Ruscio, A. M., Stein, D. J., Chiu, W. T., & Kessler, R. C. (2008). The epidemiology of obsessive–compulsive disorder in the national comorbidity survey replication. Molecular Psychiatry, 15(1), 53–63. 10.1038/mp.2008.94. 18725912 PMC2797569

[ref38] Seow, T. X. F., & Gillan, C. M. (2020). Transdiagnostic phenotyping reveals a host of metacognitive deficits implicated in compulsivity. Scientific Reports, 10(1), 1–11. 10.1038/s41598-020-59646-4. 32076008 PMC7031252

[ref39] Seow, T. X. F., Rouault, M., Gillan, C. M., & Fleming, S. M. (2021). How local and global metacognition shape mental health. Biological Psychiatry, 90(7), 436–446. 10.1016/j.biopsych.2021.05.013. 34334187

[ref40] Sheehan, D. V., Lecrubier, Y., Sheehan, K. H., Amorim, P., Janavs, J., Weiller, E., … Dunbar, G. C. (1998). The Mini-International Neuropsychiatric Interview (M.I.N.I.): The development and validation of a structured diagnostic psychiatric interview for DSM-IV and ICD-10. Journal of Clinical Psychiatry, 59(20), 22–33. 10.1016/S0924-9338(99)80239-9. 9881538

[ref41] Singmann, H., Bolker, B., & Westfall, J. (2015). Analysis of Factorial Experiments, package “afex,” 1–44. Retrieved from http://cran.r-project.org/package=afex.

[ref42] Stone, C., Mattingley, J. B., & Rangelov, D. (2022). On second thoughts: Changes of mind in decision-making. Trends in Cognitive Sciences, 26(5), 419–431. 10.1016/j.tics.2022.02.004. 35279383

[ref43] Vaghi, M. M., Luyckx, F., Sule, A., Fineberg, N. A., Robbins, T. W., & De Martino, B. (2017). Compulsivity reveals a novel dissociation between action and confidence. Neuron, 96(2), 348–354. 10.1016/j.neuron.2017.09.006. 28965997 PMC5643443

[ref44] Vanes, L. D., & Dolan, R. J. (2021). Transdiagnostic neuroimaging markers of psychiatric risk: A narrative review. NeuroImage: Clinical, 30(November 2020), 102634. 10.1016/j.nicl.2021.102634. 33780864 PMC8022867

[ref45] Wells, A., & Cartwright-Hatton, S. (2004). A short form of the metacognitions questionnaire: Properties of the MCQ-30. Behaviour Research and Therapy, 42(4), 385–396. 10.1016/S0005-7967(03)00147-5. 14998733

[ref46] Wells, A., & Papageorgiou, C. (1998). Relationships between worry, obsessive–compulsive symptoms and meta-cognitive beliefs. Behaviour Research and Therapy, 36(9), 899–913. 10.1016/S0005-7967(98)00070-9. 9701864

[ref47] Williams, N. (2014). The GAD-7 questionnaire. Occupational Medicine, 64(3), 224. 10.1093/occmed/kqt161. 25281578

[ref48] Zung, W. W. K. (1965). A self-rating depression scale. Archives of General Psychiatry, 12(1), 63–70. 10.1001/ARCHPSYC.1965.01720310065008. 14221692

