# Supplementary material for: Differences in metacognitive functioning between obsessive–compulsive disorder patients and highly compulsive individuals from the general population
Source: Psychol Med. 2023 Aug 9;53(16):7933–42. doi: 10.1017/S003329172300209X (PMC10755250; doi:10.1017/S003329172300209X)
Supplement: Hoven et al. supplementary material [file S003329172300209Xsup001.pdf]

Supplementary Information

Differences in metacognitive functioning between obsessive-compulsive disorder patients and highly compulsive individuals from the general population.

Monja Hoven*[1], Marion Rouault[2,3], Ruth van Holst[1], Judy Luigjes[1]

1 Department of Psychiatry, Amsterdam UMC, University of Amsterdam, Amsterdam, The Netherlands

2 Motivation, Brain & Behavior (MBB) lab, Paris Brain Institute (ICM), Hôpital de la Pitié-Salpêtrière, Paris, France.

3 Département d'Études Cognitives, École Normale Supérieure, Université Paris Sciences & Lettres (PSL University), Paris, France

***** *Corresponding author*

Correlations between questionnaire scores

	RSES	OCI-R	MCQ-30	DASS	ZUNGDEP	GAD-7
ASA	.77***	-.47***	-.65***	-.75***	-.55***	-.58***
RSES		-.54***	-.66***	-.71***	-.60***	-.68***
OCI-R			.72***	.76***	.53***	.35*
MCQ-30				.80***	-	-
DASS					-	-
ZUNGDEP						.66***

Supplementary Table 1: Correlations between questionnaire scores. Spearman correlation coefficients between questionnaire scores. For the correlations between rSES, OCI-R and ASA, all 120 subjects were included. For the correlations including MCQ-30 and DASS, only the 80 participants in the OCD and HC group were included. For the correlations including the ZungDEP and GAD-7 questionnaires only the HComp group was included. Abbreviations: OCD = Obsessive-Compulsive Disorder, HCs = Healthy Controls, HComp = High-Compulsive subjects, OCI-R: Obsessive-Compulsive Inventory-Revised, ASA: Autonomy Scale Amsterdam, rSES: Rosenberg Self-Esteem Scale, DASS: Depression Anxiety and Stress Scale, GAD-7: Generalized Anxiety Disorder-7 Questionnaire, ZungDEP: Zung's Depression scale, * = p<.05, ** = p<.01, *** = p<.001.

Comparing highly compulsive subjects to healthy controls

The healthy control (HC) group performed significantly better on the task compared to the highly compulsive (HComp) group, and were also significantly more confident at both local and global levels (Supplementary Table 2). Even though the HComp group had higher local and global calibration values, and both groups showed overconfidence, this did not differ significantly between the groups. Also, no group differences in discrimination or the correlation between local and global confidence were found. Together, this shows that the HComp group is just as overconfident in their abilities as the HC group, even though the HComp group performs worse and is less confident overall.

	HCs (N = 40)	HComp (N=40)	HC vs. HComp
**Age in years**	38.58 (11.11)	36.53 (12.73)	T = 0.77<br>P = 0.45
**Females (%)**	27 (67.5%)	28 (70%)	X2 = 0.06<br>P = 0.81
**Years of education**	10.20 (3.13)	10.35 (2.64)	T = -0.23<br>P = 0.82
**OCI-R**	2.90 (2.48)	23.35 (13.18)	T = -9.65<br>P < .001
**ASA**	168.13 (19.18)	160.35 (33.99)	T = 1.26<br>P = 0.21
**rSES**	23.48 (3.94)	18.53 (7.56)	T = 3.67<br>P < .001
**Accuracy (percent correct)**	76.49 (7.76)	69.90 (8.64)	F = 13.15<br>P < 0.001
**Local Confidence (on 50-100 scale)**	81.14 (8.11)	76.82 (9.58)	F = 4.76<br>P = 0.032
**Global Confidence**	80.69 (7.27)	76.21 (8.83)	F = 6.08<br>P = 0.016
**Local Calibration**	4.82 (8.92)	6.63 (11.22)	T = 0.80<br>P = 0.429
**Global Calibration**	4.20 (6.98)	6.31<br>(9.62)	T = 1.13<br>P = 0.264
**Correlation Local & Global Confidence**	0.56	0.52	T = -0.60<br>P = 0.552
**Discrimination**	8.34 (4.77)	6.73<br>(4.66)	T = -1.53<br>P = 0.130

Supplementary Table 2: Differences in demographics, clinical data and task performance between HC and HComp groups. Abbreviations: HCs = Healthy Controls, HComp = High-Compulsive subjects, OCI-R: Obsessive-Compulsive Inventory-Revised, ASA: Autonomy Scale Amsterdam, rSES: Rosenberg Self-Esteem Scale, T = T-value from two-sample t-test, F = F-value from ANOVA, P = P-value. Data are reported as mean (standard deviation).

Comparing OCD and HC groups while controlling for anxiety and depression symptoms

**Dependent Variable**	**Intercept**	**DASS score**	**Group (OCD)**
Local confidence	β = 82.193<br>SE = 1.659<br>T = 49.533<br>P < .001	β = 1.391<br>SE = 1.401<br>T = 0.993<br>P = 0.324	β = -8.508<br>SE = 2.785<br>T = -3.055<br>P = 0.003
Global confidence	β = 81.480<br>SE = 1.505<br>T = 54.141<br>P < .001	β = 1.041<br>SE = 1.271<br>T = 0.819<br>P = 0.415	β = -6.027<br>SE = 2.526<br>T = -2.386<br>P = 0.020
Local calibration	β = 7.872<br>SE = 2.098<br>T = 3.753<br>P < .001	β = 4.030<br>SE = 1.772<br>T = 2.275<br>P = 0.026	β = -11.091<br>SE = 3.521<br>T = -3.150<br>P = 0.002
Global calibration	β = 6.315<br>SE = 1.604<br>T = 3.938<br>P < .001	β = 2.798<br>SE = 1.354<br>T = 2.066<br>P = 0.042	β = -7.234<br>SE = 2.691<br>T = -2.688<br>P = 0.009

Supplementary Table 3: Regression results from models comparing OCD and HC groups while controlling for anxiety and depression symptoms. Abbreviations: HC = Healthy Controls, OCD = Obsessive compulsive disorder, DASS: Depression Anxiety Stress Scale, SE = Standard Error, T = T-value, P = P-value.

Comparing the effect of OCI-R score on local confidence between OCD and HComp groups

**Local Confidence ~**	Intercept	OCI-R score	Group (HComp)	Group (HComp) x OCI-R score
β	74.724	-2.508	2.086	4.034
SE	1.388	1.698	1.963	2.087
T	53.830	- 1.477	1.063	1.933
P	<.001	0.144	0.291	0.057

Supplementary Table 4: Regression results from model comparing the effects of OCI-R score on local confidence between OCD and HComp groups. Abbreviations: HComp = Highly compulsive subjects, OCD = Obsessive compulsive disorder, OCI-R: Obsessive-Compulsive Inventory-Revised, SE = Standard Error, T = T-value, P = P-value.

Comparing M-Ratio (metacognitive efficiency) between groups

For the sake of completeness, we calculated metacognitive efficiency for each participant. The signal detection theory framework assumes constant signal strength, and therefore metacognitive efficiency (i.e. M-Ratio) was calculated separately for the easy and hard trials (36 trials per subject per M-Ratio calculation). The M-Ratio was taken as the average M-Ratio over the easy and hard condition, and compared between groups using two-sample t-tests. Some subjects (8 OCD, 2 HC, 6 HComp) with a negative M-Ratio likely due to the low number of trials to estimate M-Ratio, were excluded for these analyses.

The average M-Ratio for OCD patients was 0.859, for HC it was 0.927, and for Hcomp it was 1.11. There were no differences in M-Ratio between the OCD and HC groups (t_{68} = 0.487, p = 0.628), and neither between the OCD and HComp groups (t_{64} = 1.136, p = 0.260).
